# What is the best strategy for the prevention of transfusion-transmitted malaria in sub-Saharan African countries where malaria is endemic?

**DOI:** 10.1186/1475-2875-12-465

**Published:** 2013-12-28

**Authors:** Jobert Richie N Nansseu, Jean Jacques N Noubiap, Shalom Tchokfe Ndoula, Albert Frank M Zeh, Chavely Gwladys Monamele

**Affiliations:** 1 Sickle Cell Care Unit, Mother and Child Centre, Chantal Biya Foundation, Yaoundé, Cameroon; 2 Internal Medicine Unit, Edéa Regional Hospital, PO Box 100, Edéa, Cameroon; 3 Guidiguis Health District, Guidiguis, Cameroon; 4 Paediatric Medicine Unit, Ebolowa Regional Hospital, Ebolowa, Cameroon; 5 Faculty of Medicine and Biomedical Sciences, University of Yaounde I, Yaounde, Cameroon; 6 Chantal Biya International Research Centre, Yaounde, Cameroon

**Keywords:** Malaria, Blood transfusion, Transfusion-transmitted malaria, Sub-Saharan Africa

## Abstract

The transmission of malaria by blood transfusion was one of the first recorded incidents of transfusion-transmitted infections (TTIs). Although the World Health Organization (WHO) recommends that blood for transfusion should be screened for TTIs, malaria screening is not performed in most malaria-endemic countries in sub-Saharan Africa (SSA). The transfusion of infected red blood cells may lead to severe post-transfusion clinical manifestations of malaria, which could be rapidly fatal. Ensuring that blood supply in endemic countries is free from malaria is highly problematical, as most of the donors may potentially harbour low levels of malaria parasites. Pre-transfusion screening within endemic settings has been identified as a cost-effective option for prevention of transfusion-transmitted malaria (TTM). But currently, there is no screening method that is practical, affordable and suitably sensitive for use by blood banks in SSA. Even if this method was available, rejection of malaria-positive donors would considerably jeopardize the blood supply and increase morbidity and mortality, especially among pregnant women and children who top the scale of blood transfusion users in SSA. In this context, the systematic prophylaxis of recipients with anti-malarials could constitute a good alternative, as it prevents any deferral of donor units as well as the occurrence of TTM. With the on-going programme, namely the Affordable Medicine Facility - Malaria, there is an increase in the availability of low-priced artemisinin-based combination therapy that can be used for systematic prophylaxis. It appears nonetheless an urgent need to conduct cost-benefit studies in order to evaluate each of the TTM preventive methods. This approach could permit the design and implementation of an evidence-based measure of TTM prevention in SSA, advocating thereby its widespread use in the region.

## Background

Malaria is a protozoan parasitic infection of humans resulting from one or more of the five species of the genus *Plasmodium* (*Plasmodium falciparum, Plasmodium vivax, Plasmodium ovale, Plasmodium malariae* and *Plasmodium knowlesi*) [1]. It is one of the most important parasitic diseases in the world and remains a major challenge to mankind. Malaria can be efficiently transmitted by transfusion of cellular blood components and it is undoubtedly responsible for the majority of transfusion-transmitted diseases in the world [2,3]. But, mainly due to the high interest in human immunodeficiency virus blood safety, transfusion transmission of malaria has been a neglected topic until recently [3,4]. As a result, there has been a paucity of information concerning the distribution and potential role of the different *Plasmodium* species in transfusion-related malaria cases, and the clinical impact of parasitaemic blood in recipients, particularly young children and pregnant women who are the highest consumers of blood transfusions in sub-Saharan Africa (SSA) [4,5]. When malaria is transmitted through blood transfusion to a non-immune recipient, it can progress rapidly and may lead to significant morbidity and mortality, specifically when diagnosis is delayed [1,3].

The incidence of transfusion-transmitted malaria (TTM) among people residing in endemic areas is unknown. As a matter of fact, a substantial proportion of the population in malaria-endemic countries has asymptomatic parasitaemia, making it difficult to be sure whether malaria occurring after blood transfusion was acquired from the transfusion or not [3,6]. Nonetheless, the World Health Organization (WHO) recommends that all blood donations should be screened for malaria where “appropriate and possible”, and that there should be quality assured testing for transfusion-transmitted infections (TTIs) [7]. These recommendations have significant resource implications and have not been widely implemented by transfusion services in SSA [3,5]. Indeed, there are reasons for the difficulty in screening blood for malaria in SSA. Severe blood shortages are widespread and would be exacerbated by rejecting blood that contains malaria parasites [3]. More so, there is currently no assay to screen blood with low-levels of parasites that is sensitive, practical and affordable enough for use by transfusion services in endemic countries [3,8]. Hence, there is no evidence-based guidance to indicate which malaria screening methods are effective for use by transfusion services in malaria-endemic countries or what action should be undertaken if the donated blood tests positive [3].

Other transfusion guidelines suggest that transfusion recipients should be given systematic anti-malarial prophylaxis [3,5,6]. For many years, presumptive anti-malarial treatment with inexpensive chloroquine was given to blood recipients to prevent TTM [1,2]. However, the spread of chloroquine resistance across SSA has led to such a strategy becoming redundant and ineffective [1,2]. Alternatives to chloroquine, such as artemisinin and artemisinin-based combination therapy (ACT) are considerably more expensive, weakening the applicability and usefulness of anti-malarial prophylaxis in resource-poor settings until latterly [2,3].

Fortunately, a new programme, the Affordable Medicines Facility - Malaria (AMFm), has recently been put in place with satisfactory results. This is a pilot supra-national subsidy programme that aims to increase access and affordability, therefore, reducing the price of ACT to levels similar to that of less effective anti-malarials (such as sulphadoxine-pyrimethamine and chloroquine). The evaluation of this programme shows that there is an increased availability of low-priced ACT with no significant variation in availability based on remoteness [4]. Its implementation in SSA could thereby reinforce the use of ACT for systematic prophylaxis of blood recipients in order to efficiently prevent TTM as well as an unnecessary wastage of blood units, even in remote areas. The present review aims to highlight the burden of TTM in SSA, and discusses the strategies for the prevention of TTM in these countries.

### An overview of the burden of malaria in sub-Saharan Africa

Malaria remains a major public health hazard in SSA owing to its high morbidity and mortality despite being the focus of significant financial support and research. According to the latest World Malaria Report summarizing data received from 104 malaria-endemic countries and territories, there were about 219 million cases of malaria and an estimated 660,000 deaths, 90% of which occurring in SSA [5].

Malaria exacts a heavy toll of illness and death among children specifically the under fives, and on pregnant women [5]. In fact, most children in endemic areas experience their first malaria infections during the first two years of life, when they have not yet acquired adequate immunity, which makes these early years of highest risk. Ninety per cent of all malaria deaths in Africa occur in young children [6]. A child dies every 45 seconds as a result of malaria, the disease accounting for 20% of all childhood deaths [5].

Malaria in pregnancy is widespread. Pregnant women are highly vulnerable because of iron deficiency, a major problem in malaria-endemic areas. It endangers the health of women as well as that of the newborn. Malaria causes anaemia and low birth weight as a consequence of the loss of previously existing immunity [7]. Malaria accounts for 6.5% of abortions, 15% of premature deliveries and 0.7% of deaths *in utero*[8]. An estimated 200,000 infants die annually as a result of malaria infection during pregnancy [5].

A contributing factor to the malaria problem in SSA is the diversity of the parasite that infects humans. Five species infect man of which *P. falciparum* is the most virulent [8]. These malaria parasites can develop within, invade red blood cells (erythrocytes) and consume up to 75-80% of their haemoglobin as nutrient source [6]. *Plasmodium falciparum* causes severe complications, such as cerebral malaria, severe anaemia, acute renal failure, hypoglycaemia and pulmonary infection [7]. Severe anaemia will occur when the parasite disrupts the erythrocytes, giving rise to the necessity of blood transfusion.

### Blood transfusion: a life-saving but risky intervention

Every second, someone in the world needs blood [9]. In every country, surgery, trauma, severe anaemia, and complications of pregnancy are among clinical conditions that demand blood transfusion [9]. Whatever the degree of development of a health care system, transfusion is the only option for survival for many patients. An adequate supply of blood is essential for reducing mortality and morbidity in SSA, especially among young children and pregnant women [10], but critical shortages are common. For instance, in SSA, 26% of maternal haemorrhage-related deaths during the period 1970-2007 were due to lack of available blood for transfusion [11].

Many patients, particularly in SSA, do not have access to blood when they need it. Of the estimated 80 million units of blood donated annually worldwide, only 38% are collected in the developing world where 82% of the world’s population lives [9]. Up to 150,000 pregnancy-related deaths could be avoided each year through access to safe blood [9]. Moreover, anaemia as a result of malaria accounts for 70% of all blood transfusions given to children in SSA [12]. Many children die whilst waiting for transfusion [12,13]. A Kenyan study found, for instance, that over 60% of children in need of transfusion may die while waiting for a blood donor [12]. What’s more, in some Ghanaian regions, nearly 33% of all blood transfusions given are to infants aged below 3 years [14].

Despite the fact that blood transfusion can be life-saving, there are associated risks, particularly the transmission of blood-borne infections. Screening for TTIs, to exclude blood donations at risk of transmitting infection from donors to recipients, is a critical part of the process of ensuring that transfusion is as safe as possible. Effective screening for evidence of the presence of the most common and dangerous TTIs can reduce the risk of transmission to very low levels [15]. According to WHO, regular, voluntary, non-remunerated blood donors from low-risk populations are the foundation of a safe blood supply [9]. Blood transfusion services should therefore establish efficient systems to ensure that all donated blood is correctly screened for specific TTIs and that only non-reactive blood and blood components are released for clinical and manufacturing use [16].

To be transmissible by blood, the infectious agent must have the following characteristics: (i) presence in the blood for long periods, sometimes in high titers, (ii) stability in blood stored at +4°C or lower, (iii) long incubation period before the appearance of clinical signs, and (iv) asymptomatic phase or only mild symptoms in the blood donor, hence not identifiable during the blood donor selection process [17]. *Plasmodium* species respond to all the aforementioned criteria [16]. Malaria can thus be efficiently transmitted by transfusion of cellular blood components.

### Prevalence and severity of transfusion–transmitted malaria

Batista-dos-Santos *et al.* reported that the history of TTM dates back to 1882, when Gerhardt empirically demonstrated the transmission of malaria in humans by infected blood [18]. However, the first case of accidental transmission of malaria by blood transfusion was described in 1911 [18]. Worldwide, around 3,000 cases of TTM were reported between 1950 and 1980. These were predominantly from non-endemic countries, so this is believed to be a significant underestimate of the global burden [19].

The prevalence of malaria parasitaemia in African donors depends on the local endemicity and transmission season and varies from 0.67% in Nairobi, Kenya, a non-endemic area, to over 55% in highly endemic northern Nigeria (with a median prevalence of 10.2%) [20]. *Plasmodium* species are the most prevalent transfusion transmissible pathogens especially in SSA [20]. In fact, among SSA blood donors, the prevalence of human immunodeficiency virus, hepatitis C virus and hepatitis B virus ranges from 0.5-16%, 0.5%-12.3% and 2.5-20% respectively [21].

Given that asymptomatic carriage of malaria parasites is common in malaria-endemic countries [22], parasitaemia detected in a blood recipient could have been acquired from a mosquito bite rather than from the transfused blood. TTM can consequently only be confirmed by genotyping to demonstrate that the parasite in the recipient is identical to the one in the transfused blood. Studies show that the frequency of post-transfusion malaria varies from less than 0.2 cases per million recipients in non-endemic countries to 50 per million in endemic ones [23].

Anecdotally, Owusu-Ofori *et al.*[24] conducted a study, the result of which showed a genotypically confirmed TTM in only one (2%) of their 50 patients who received a blood transfusion positive for *P. falciparum* by polymerase chain reaction (PCR). The parasite density in the blood unit that caused the TTM was 280/μL [24]. Another study from Sudan without performing PCR testing and genotyping, found that all 12 patients (100%) who received malaria-positive blood developed microscopically confirmed malaria. But the same study also revealed that two patients (0.52%) developed parasitaemia after transfusion despite receiving microscopy-negative blood [25], claiming accordingly that not all cases of malaria occurring after transfusion are transmitted by that transfusion. This last finding has to be taken with caution, given that without performing PCR testing, the two blood units could have falsely been declared negative.

Transmission of malaria has been reported to occur mainly from single-donor products [26]. Any blood component may harbour viable parasites. Ninety-five per cent of transfusions in SSA involve whole blood rather than components [20]. Whole blood and concentrated erythrocytes represent the most common sources of TTM; however, some cases of TTM have also been reported after transfusing platelet concentrates, leukocyte concentrates, cryoprecipitate (contaminated by residual erythrocytes), and frozen erythrocytes after thawing and washing. Conversely, transmission through freshly frozen plasma has not been reported, even though this product is not of common use in SSA [27,28].

Additionally, in cases of TTM, depending on the number of parasites in the inoculum, the symptoms of malaria may begin days or weeks after transfusion [29], presenting as a serious and often fatal disease [30], especially for non-immune recipients [31]. For instance, as reported by Freimans *et al.*, as few as ten parasites are sufficient to initiate fulminant malaria in humans [1].

The majority of recipients of blood transfusions living in malaria-endemic areas are semi-immune to malaria [32], but the degree of protection that this immunity confers against TTM is unknown [20]. Young infants in areas where malaria is endemic who have not had repeated exposure to the parasite may be regarded as non-immune recipients [20] and as a consequence, they may be as susceptible to TTM as a non-immune person who lives in a non malaria-endemic country. Besides, pregnant women and immunocompromized patients, who with young children are the biggest demanders of blood transfusions in Africa, may also be at a high risk of TTM [24]. The clinical severity of TTM is likely to be very different in malaria-endemic countries compared to non-endemic ones [20].

The risk of TTM has been associated with the difficulty in identifying infected potential donors, mostly those with a low number of parasites circulating in their blood, as well as the ability of this parasite to remain viable in stored blood units, even after the storage process [27,28]. Thus, the transfusion practice constitutes a major challenge in malaria-endemic areas because many potential blood donors are infected. This situation could jeopardize the attainment of the huge demand for transfusion of blood and blood products in areas where refusal of donation is high, and donation shortages are frequent [27]. Only a few parasites in a unit of blood are sufficient to cause infections in susceptible individuals namely children, pregnant women and immunocompromized patients [20]. TTM presents, therefore, a public health risk, requiring effective methods of donor screening [33]. Any malaria screening test used by the transfusion services in SSA needs to be highly sensitive [20,34].

### Screening for transfusion-transmitted malaria

The ability to screen blood donations, as well as donors, can significantly decrease any risk of TTM [27]. Laboratory screening for malaria remains the possible option for reducing transfusion malaria [23]. There are four specific targets for donation screening: intracellular parasites, plasmodial antibodies, plasmodial antigen, and plasmodial DNA [27].

In routine practice, the “gold-standard” technique, optical microscopy in thick blood smears, is the most often used for *Plasmodium* detection in malaria-endemic areas [18]. This technique is considered the most effective and inexpensive for the diagnosis of malaria [18]. Its sensitivity varies depending on the expertise of the microscopist. In experienced hands, sensitivities of 5-50 parasites/μL can be achieved, but in routine practice most laboratories achieve a lower sensitivity of around 500 parasites/μL [35,36]. Further, a single parasite identified by microscopic evaluation of a thick blood film (4 mL) is equivalent to almost 10,000 parasites in a 450 mL unit of blood [20]. But despite their continued application as key diagnostic tests, microscopy techniques have some major limitations that render them inappropriate for universal or targeted donor screening. Precisely, they lack the required sensitivity and specificity to detect all infected units, specifically in situations of low parasite density, hence presenting a real transfusion risk for the recipient [18,27]. In addition, they are time-consuming (generally requiring one hour or more for preparation and detailed examination), are inadequate for examining a large volume of samples, and do require considerable expertise and specialized equipment when fluorescent methods are used [27,37,38]. This hinders a rapid evaluation, particularly in a blood transfusion service. Finally, post-transfusion malaria cases have been reported in recipients of blood that has been tested negative by microscopy [25]. Microscopy detection of malaria parasites is consequently likely to significantly underestimate the prevalence of parasitaemia in blood donations, and appears not sensitive enough to be recommended as the suitable screening test for transfusion services in SSA malaria-endemic settings [20].

Alternative methods have been developed for the screening of malaria for use both in areas where malaria is endemic and in areas where it is not, detecting specific *Plasmodium* antigens or antibodies directed against the *Plasmodium*. The detection of malarial antigen with rapid diagnostic tests (RDTs) was originally intended as a more rapid and objective alternative to direct microscopy [27]. RDTs detect *Plasmodium*-specific parasite proteins, such as pan-malarial lactate dehydrogenase (pLDH), and *P. falciparum* specific histidine-rich protein 2 (HRP2). Most of these assays are in a ‘dipstick’ format that can be used with minimal training, are field applicable, and provide a result within 10-20 minutes [27,33,39]. However, RDTs methods have not offered improved sensitivity over microscopy, and their sensitivity decreases as parasitaemia falls below 100 parasites/μL. Also, false positives are observed, especially after treatment, as the parasite antigens detected can remain in the circulation following parasite clearance, this being especially the case for the HRP2 antigen-based tests. Eventually, current RDTs are either specific to *P. falciparum*, or they cannot distinguish between the parasite species present [33,35]. Heutmekers *et al.* using a RDT CareStart pLDH showed for instance that overall sensitivity for *P. falciparum* and *P. vivax* was good, but poor for *P. ovale* and *P. malariae*[40]. In order to increase the likelihood to detect all types of *Plasmodium species*, it has been proposed a combined HRP2/pLDH-based RDT [41]. Heutmekers *et al.* showed on another hand that false-negative results mainly occurred at parasite density less than 100/μL [40]. Atchade *et al.* claimed that the pLDH-based RDT can exhibit a detectability threshold of 1 parasite/μL, lower than that of the other methods, with the exception of PCR, and that unlike HRP2-based tests, false positives are exceptional with pLDH-based RDTs [42]. Based on these findings, the pLDH antigen detection for *Plasmodium species* could be an interesting tool for blood donation qualification in order to ensure blood safety in malaria-endemic areas [30,42]. It has been noticed nonetheless that seasons influenced pLDH prevalence. What’s more, if the donors had taken self-treatment measures prior to blood donation such as drugs or herbal teas, malaria infection was masked and pLDH detection failed [42].

Following infection with *Plasmodium species*, the immune response results in the formation of specific antibodies not necessarily protective, and not necessarily indicating that the person is harbouring malaria parasites as well [43]. Antibody detection assays demonstrate high antibody levels and good sensitivity in semi-immune individuals, the very donors who are potentially at high risk of acting as a source of TTM by being asymptomatic but parasitaemic [43]. After having compared the IFAT assay with the DiaMed ELISA malaria antibody test, Ehghouzzi *et al.* showed that the latter was more sensitive and specific than the former, with a possibility of automation, fulfilling thereby the criteria of a satisfactory and reliable malaria screening test [44]. However, a negative malarial antibody test cannot guarantee that the donor is not infected with malaria parasites, as the antibodies may not be detectable in the first few days of malarial illness, and infection with *P. ovale* and *P. malariae* may not be detected by *P. falciparum* and *P. vivax* antigen-based assays [43]. Given the potential for malaria parasites to persist in certain patients for some years, it is worth noting that in individuals who have suffered repeated attacks of malaria, anti-malarial immunoglobulins may be detectable for several years. Even though the persistence of antibodies long after cure of the malarial infection would lead to some individuals, who are no longer parasitaemic, to be excluded as potential blood donors, it does provide a useful margin of safety if candidate donors, who are malaria-antibody positive, are excluded from donating [43]. Malaria antibody tests are therefore useful in non-endemic areas where they will result in rejecting blood donation in case of positivity, but these assays are of no use in malaria endemic areas. Indeed, malaria antibody prevalence is very high: 87% in Benin, and 65.33% in Senegal [30,42]. This would lead to a high rate of blood donor deferral as most of the populations in endemic zones harbour anti-malarial immunoglobulins.

Methods based on molecular biology have been used to detect different types of *Plasmodium* by PCR, such as the nested PCR [45]. This technique is based on the amplification of a fragment of the small subunit ribosomal RNA of the parasite and has been used for the diagnosis of malaria for research purpose and reference laboratories [38,46,47]. The PCR technique can detect parasites below the threshold levels of microscopy. Indeed, when performed under optimal conditions, PCR can detect parasitaemia as low as 0.004 to 1 parasite/μL of blood [27,46,47]. However, the result directly depends on the quality of the genetic material (DNA) of the parasite obtained during extraction and amplification, and on the quality of the reagents. Furthermore, the test is very expensive, requires extensive training and a long analysis time [18,23,27], restricting thereby its usage as a routine diagnostic test for malaria in SSA laboratories or blood banks [38,47-49]. Real-time PCR is considered at the moment to be the best molecular biology technique for the diagnosis of malaria [18,38,48,50]. It prevents ambiguous results because it does not require agarose gels, minimizes manual work, reduces pipetting errors, performs well under high throughput, and provides quantitative results of parasite density [38]. Batista-dos-Santos *et al.* have described real time PCR as a necessary, appropriate and inexpensive method, with higher sensitivity and specificity compared to those previously described, which can be adopted as part of the laboratory screening in haemotherapy centres, especially in malaria-endemic areas [18]. Owing to its prohibitive cost as well as the fact that the infrastructure needed is scarcely available in SSA malaria-endemic zones, which by the way are almost all resource-limited settings, PCR is not currently, or in the foreseeable future, a viable alternative for the screening of blood donations [27]. PCR may be probably best used in a stepwise fashion when other testing modalities are non-diagnostic but when the index of suspicion for malaria is high [33].

A recently available technique based on detection of haemozoin pigment in white blood cells by automated haematology cell counters has been described as a convenient, less costly and objective malaria screening method [23,51]. According to the review from Campuzano-Zaluaga *et al.*, the accuracy for malaria diagnosis using automated haematology analysis may vary according to species, parasite load, immunity and clinical context where the method is applied [51]. Its overall sensitivity ranges from 48.6% to 100%, and its specificity, from 25.3% to 100%. The sensitivity has been shown to decrease down to 50% with parasitaemia of less than 0.1%. Another factor tempering automated haematology analysis utilization is that laboratory staff ought to receive appropriate and continuous training allowing them to recognize malaria-related changes during validation of cell blood count results [51].

Apart from laboratory screening, donor questioning has also been proposed as another tool for malaria screening in order to lessen the risk of TTM. It aims at deferring all potential blood donors who have experienced a febrile episode at least three months before blood donation. But this strategy lacks the capacity of eliminating asymptomatic but malaria-parasitaemic blood donors [30,42]. To be useful, the medical selection through donor questionnaire must be integrated in an algorithm including other screening tools [33].

Overall, a number of factors need to be considered in selecting the most appropriate assays. In general, a balance has to be found between screening needs and the resources available, including finances, staff and their level of expertise, equipments, consumables, and disposables [16]. Each screening system has its advantages and limitations that should be taken into consideration when selecting assays. Some limitations include: (i) the length of time following infection before the screening test becomes reactive (window period), (ii) rates of biological false positives which may result in the wastage of donations and unnecessary deferral of donors, and (iii) the complexity of some systems that require automation [16].

According to WHO guidelines, the minimum evaluated sensitivity and specificity levels of all assays used for blood screening should be as high as possible and preferably not less than 99.5% [16]. But, we have seen from what precedes that the sensitivity of the most currently used methods for malaria detection in SSA blood units is much lower than this required threshold, so as to detect level of parasitaemia capable of causing TTM (approximately 0.00004 parasites/μL or 1-10 parasites/unit of blood) [20]. In fact, Owusu-Ofori *et al.* after performing thick blood films, RDT, enzyme immunoassays and real time PCR for malaria screening of blood donor units concluded that none of these four tests would be ideal for African blood banks to be used for the prevention of TTM, as they were either insufficiently sensitive or too sensitive for malaria parasites detection in blood donor units [24].

### Pre-transfusion screening of donor blood units and systematic anti-malarial prophylaxis for recipients: which one is the most cost-effective strategy to lessen the risk of transfusion-transmitted malaria in sub-Saharan Africa?

The transfusion practice constitutes a major challenge in malaria-endemic areas because many potential blood donors are infected. Identifying low-risk individuals is virtually impossible. International policies recommend that all blood donations should be screened for malaria where “appropriate and possible [16], but this is not of routine practice in SSA. It has clearly been figured out that routine screening of all donated blood would efficiently prevent infected blood donations especially when the risk of TTM appears relatively high, hence a reduction in *Plasmodium* transmission particularly in critical patients, such as children and pregnant women [42,52].

A good screening tool for malaria detection in blood units must have a high sensitivity, a high positive predictive value; it must be capable of recognizing all the *Plasmodium* species, must be rapid and less costly. Moreover, it must enable a reduction of TTM risk as well as that of falsely deferred blood donors [29,30]. But, to date, there is no test to screen blood units for low-level parasitaemia that is sensitive, practical and affordable enough for use by transfusion services in endemic countries [2,20,24]. Some test are insufficiently sensitive (optical microscopy and RDTs) while others are too sensitive or cost-prohibitive (antibody detection tests and PCR) [20]. It is true nonetheless that pLDH-based RDTs have been advocated as a valid tool for malaria screening in blood banks of malaria-endemic settings, as they were able to detect at least one parasite/μL [30,42]. But there has not been any cost-benefit analysis to support this recommendation, and Ansah *et al.* found that it was less clear if replacing microscopy with RDT-based diagnostics should be recommended on cost-effectiveness grounds [39].

So, there is not any current “realistic” and suitable method to screen for malaria parasites in blood units. And even if this method was available, there is no evidence-based policy indicating what action should be taken if the donated blood is tested positive, bearing in mind that in some regions almost 55% of blood units contain malaria parasites [20]. Thus, rejection of malaria-positive donors would substantially jeopardize the blood supply in a context where critical shortages and refusal of blood donations are common [9-11,42]. After proposing pLDH as a suitable tool for malaria screening, Atchade *et al.* note however that the feasibility of rejecting positive blood donations with regard to blood availability in malaria-endemic areas remains a serious problem [42]. Such rejection will result undoubtedly in an increased morbidity and mortality, predominantly among women and children [16]. For instance, Rajab *et al.* have shown that if malaria-positive donor units were to be excluded from the Kenyan national blood supply, an estimated 5% of blood units (compared to 1.3% for HIV, 3.6% for hepatitis B virus and 1.3% for hepatitis C virus) would be wasted [23]. The wastage would be more enormous in northern Nigeria where the prevalence of malaria in blood donations reaches almost 55% [20].

Pre-transfusion screening will lead thus to the detection of malaria-positive blood units that will be afterwards unfortunately rejected. In order to prevent unnecessary deferral of potential blood donors as well as rejection and substantial wastage of malaria-positive donor units, many authors, as well as WHO, advocate appropriate and effective malaria prophylaxis to be administered with every transfusion especially in highly endemic areas [2,16,20,23,24,27]. Anti-malarial prophylaxis with every donation allows for an increase in blood supply, as malaria-positive units are still available for transfusion. This prophylaxis used affordable chloroquine or sulphadoxine-pyrimethamine (SP). But since chloroquine resistance became widespread in SSA, it has recently been replaced by more expensive ACT, increasing the cost of treating all transfusion recipients with anti-malarials by almost ten-fold and making this practice cost-prohibitive and unaffordable on a wide scale [1,2,20,23].

There have been some claims that the cost per case prevented of transfusion-associated malaria is considerably higher for recipient anti-malarial prophylaxis than pre-transfusion screening using an automated flow cytometry technique [23]. Anecdotally, a cost analysis undertaken by Rajab *et al.* in 2005 showed a cost per case prevented of US$1.4 in adults and US$0.69 in paediatrics for the option of recipient prophylaxis using a SP-based drug. The cost escalated to US$7.79 in adults and US$5.84 in paediatrics if the prophylaxis was upgraded to the recommended ACT. For the option of pre-transfusion screening using an automated technique, the cost was US$0.03 [23]. However, it is notable that the initial cost of the equipment for that automated technique may appear prohibitive, and the technique is susceptible to miss early infections and may also record false positives even after parasitaemia clears [23]. Likewise, Owusu-Ofori *et al*., based on the low incidence of TTM they found, concluded that malaria transmission occurs infrequently, suggesting that routine treatment for all recipients of blood transfusion may probably be unnecessary [24] whilst considering that inappropriate drug use may consistently contribute to the appearance of drug resistance [23].

Happily, there is an emerging programme that can help to reinforce the practice of malaria prophylaxis by considerably lowering the price of ACT. This is the AMFm. It is a “factory-gated” subsidy for ACT launched in 2010 and currently being piloted in seven African countries. The goals of the AMFm are to increase the availability and reduce the prices of ACT to levels similar to that of less effective anti-malarials, and to displace artemisinin monotherapy whose widespread availability and improper use threaten to accelerate parasite resistance to artemisinin. A recent evaluation of this programme in Tanzania showed that average ACT prices for an adult dose fell from US$1.03 to 0.81 within six to eight months (this price being cheaper for paediatric doses), as well as it has led to a large increase in availability of the drugs with no significant variation in availability based on remoteness [4].

Furthermore, governments of some SSA countries such as Cameroon have decided to give ACT to children under five years old free of charge, and have subsidized adult doses. This strategy could also contribute in reinforcing malaria prophylaxis for blood recipients as an affordable policy given that most of the recipients in SSA are young children. In parallel, a recent study showed that almost 50% of blood recipients have received a malaria treatment with their blood transfusion, 84% of which were children, and in almost 80% of cases the anti-malarials were prescribed at the same time as the blood transfusion [2]. In malaria-endemic areas, anti-malarials are given presumptively on the basis that most febrile children presenting with severe anaemia have underlying malaria and that they are at high risk of TTM [2,24,27]. It appears preferable in such cases to presumptively treat the other 50% of recipients for whom the malaria treatment was not yet ordered, without performing any screening test before the transfusion.

Alternatives to donor screening for reducing the burden of TTM have been published (see Table 1), even though for most of them their implementation is cost-prohibitive. These include adding anti-malarials to the blood pack, marking units that test positive for malaria, and only screening blood destined for neonates [20]. There are also additional strategies that can be implemented, depending on the geography of the country, the periodicity of malaria (seasonal or year round), the type and age of the donors, and the age, gender and underlying condition of the recipients, together with their existing malaria status. For example, the algorithm recently proposed by Sobani *et al.* in Pakistan is mainly based on the aforementioned strategy [33]. Finally, some promising new technologies are forthcoming, such as pathogen reduction measures that have the potential to reduce TTM with *P. falciparum* being highly sensitive to inactivation by photochemical treatment with amotosalen and long wavelength ultraviolet light [20]. But this strategy is not yet implemented even in Northern countries, and its feasibility in SSA is questionable, as this technique is not cheap and requires trained personnel as well as sophisticated equipments. Should it be possible to reduce its cost, the additional benefit of preventing the transmission of other pathogens present in the African blood supply would be considerable.

**Table 1:** Summary of recommendations from published studies advocating the reduction of transfusion-transmitted malaria in malaria endemic areas

	**Recommendation**
**Screening**	Blood donation policies should incorporate malaria screening
	Donors should be screened for malaria before donation
	Blood for neonates should be screened for malaria
**Deferral/retention**	All blood infected with malaria should be rejected
	Blood screened for malaria should be retained but marked negative or positive
**Treating recipients or blood packs**	Anti-malarials should be added to donated blood to eradicate parasite *in vitro*
	Photochemical inactivation of parasites with amotosalen and long-wavelength ultraviolet light in platelet and plasma components
	All neonates should be treated after every transfusion
	All recipients of malaria-infected blood should be treated for malaria
	Presumptive treatment for all recipients

From Owusu-Ofori *et al.*[3].

However, it is worth emphasizing that despite the different strategies adopted, it is virtually impossible to safeguard the blood supply from malaria in endemic countries, and often the transfusion of blood, together with the judicial use of anti-malarial drugs, is necessary to minimize the occurrence of TTM in the recipient. In addition, promoting the appropriate clinical use of blood has a role in minimizing TTM by ensuring that patients receive blood only when needed and, therefore, are not exposed unnecessarily [27]. Finally, it appears as an urgent need to perform cost-benefit analysis of the different screening tools for malaria detection in blood units, so as to identify which method is the most cost effective to be implemented as the gold standard for malaria screening of blood units in order to lessen the risk of TTM, especially in SSA malaria-endemic regions.

## Conclusion

The critical lack of evidence about the clinical impact of TTM and the absence of an effective and feasible screening method are impediments to rational decision-making about when and how to screen blood for malaria. There are no screening tools for malaria that are practical, affordable, and suitably sensitive for use by blood banks in SSA. The prevalence of malaria in blood donors is variable but can reach 55% in some parts of West Africa. Implementation of any policy that advocates deferral of all such donors will have a significant negative impact on the availability of blood for transfusion, will undoubtedly increase mortality particularly among pregnant women and children, and must be underpinned by robust evidence. With the availability of subsidized and low-priced ACT, the anti-malarial prophylaxis with every transfusion appears a practical alternative strategy to lessen TTM in SSA. However, cost-benefit studies should be conducted to point out the most suitable method to be used for the prevention of TTM. One should however bear in mind that complete prevention of TTM may not be attainable, so malaria must always be considered in any patient with a febrile illness post-transfusion.

## Abbreviations

ACT: Artemisinin-based combination therapy; AMFm: Affordable medicines facility-malaria; DNA: Deoxyribonucleic acid; ELISA: Enzyme-linked immunosorbent assay; PCR: Polymerase chain reaction; RDT: Rapid diagnostic test for malaria; RNA: Ribonucleic acid; TTIs: Transfusion-transmissible infections; TTM: Transfusion-transmitted malaria; SSA: Sub-Saharan Africa; WHO: World Health Organization.

## Competing interests

The authors declare that they have no competing interests. They have not benefited from any sponsorship and funding.

## Authors’ contributions

JRNN conceived the review, took part in the compilation of background material, drafted and revised the manuscript. JJNN conceived the review, conducted the compilation of background material and contributed to the manuscript drafting and revision. SNT, AFZM and CGM critically reviewed and revised the manuscript. All the authors approved the final version of the manuscript.
